# Facultative Endosymbiont *Serratia symbiotica* Provides Fitness Benefits for Celery Aphid *Semiaphis heraclei* Collected from Plant *Cnidium monnieri*

**DOI:** 10.3390/plants14213391

**Published:** 2025-11-05

**Authors:** Chunyan Chang, Yingshuo Han, Kun Yang, Xin Jiang, Xinrui Zhang, Zhuo Li, Feng Ge

**Affiliations:** 1 Shandong Key Laboratory for Green Prevention and Control of Agricultural Pests, Institute of Plant Protection, Shandong Academy of Agricultural Sciences, Jinan 250100, China; changchunyan36@163.com (C.C.); zhh0277@163.com (Y.H.); 18911895763@163.com (X.J.); xy20126043@163.com (X.Z.); 2 College of Plant Health and Medicine, Qingdao Agricultural University, Qingdao 266109, China; yangkun@qau.edu.cn

**Keywords:** *Semiaphis heraclei*, *Serratia symbiotica*, aphid fitness, developmental duration, tripartite interaction

## Abstract

*Semiaphis heraclei* Takahashi (Hemiptera: Aphididae) serves as a vital resource for natural enemies from functional plant *Cnidium monnieri* (L.) Cusson (Apiaceae), playing a crucial role in ecological dynamics. Endosymbionts influence the performance of their hosts. Here, we determined the communities of facultative endosymbionts in aphids from *Lonicera japonica* Thunb. (Caprifoliaceae), *Apium graveolens* L. (Apiaceae), and *C. monnieri* and assessed the performance of four aphid clones. The infection rates of *Serratia symbiotica* Moran (Gammaproteobacteria: Enterobacteriaceae) and *Regiella insecticola* Moran (Enterobacteriales: Enterobacteriaceae) reached 100%. Notably, the infection rates of *Spiroplasma* and *Rickettsia* varied across host plants. Fitness assessment revealed that aphids performed better on their natal hosts, exhibiting shorter nymphal development times and higher fecundity. *S. symbiotica* had contrasting effects on aphids based on their origin. It prolonged the development duration and decreased the intrinsic rate of increase (*r*_m_), net reproductive rate (*R*_0_), and finite rate of increase (λ) in aphids collected from plant *A. graveolens*. However, for aphids collected from plant *C. monnieri*, it shortened the doubling time (*DT*) and improved *r*_m_, *R*_0_, and λ, while prolonging the mean generation time. Our studies are the first to investigate the infection status and role of facultative endosymbionts in aphid *S. heraclei*, extending the documented effects of plant diversity to fluctuations in the infection rate, with potentially far-reaching consequences for related endosymbionts’ ecosystem processes.

## 1. Introduction

*Semiaphis heraclei* (Takahashi) (Hemiptera: Aphididae), a holocyclic aphid, utilizes *Lonicera* spp. as its primary host and plants from the Apiaceae family (e.g., *Apium graveolens* (L.) (Apiaceae), *Cnidium monnieri* L. Cusson (Apiaceae), *Foeniculum Vulgare* Mill (Apiaceae), etc.) as secondary hosts. It has been recorded in several provinces in China (e.g., Liaoning, Shaanxi, Hebei, Shandong, Henan, Sichuan, Hubei, Guangxi, etc.) [1,2]. The infestation of *S. heraclei* inflicts serious harm on its host plants (i.e., it can be harmful to the growth of *C. monnieri* by depleting plant phloem sap through its piercing–sucking mouthparts), causing leaf shriveling, yellowing, and withering in plants [3]. Additionally, the honeydew excreted by *S. heraclei* promotes the growth of sooty mold, seriously hindering the photosynthetic ability of the plants [4]; the transmission of plant viruses recruits more herbivores and poses a significant threat to plant health [5,6,7,8]. Aphids prefer virus- or fungal-infected plants, thereby increasing plant damage [6]. The host range of *S. heraclei* is restricted to the Caprifoliaceae and Apiaceae plants. Consequently, it does not pose a threat to grain crops (e.g., wheat, maize), economic crops (e.g., cotton, pepper), and fruit crops (e.g., apples and pear) [9,10]. Beyond its impacts as a dominant pest for *C. monnieri*, *S. heraclei* also plays a vital role in various ecological processes. It serves as a vital food source for numerous predators attracted to *C. monnieri*, such as ladybugs (e.g., *Propylaea japonica* Thunberg (Coleoptera: Coccinellidae), *Harmonia axyridis* Pallas (Coloptera: Coccinellidae)**,** and *Hippodamia variegata* Goeze (Coloptera: Coccinellidae)), hoverflies (e.g., *Episyrphus balteatus* De Geer (Diptera: Syrphidae)), and lacewings (e.g., *Chrysoperla sinica* Tjeder (Neuroptera: Chrysopidae)) [1,11], contributing to the overall diversity and stability of natural ecosystems.

As a potential functional plant, *C. monnieri* not only attracts predatory natural enemies of insect pests [9] but also serves as a food source and shelter for natural enemies and pollinators, which can migrate into crops and orchards to facilitate plant pollination and enhance biological pest control [12,13,14]. The colonization of natural enemies (e.g., ladybugs, hoverflies, lacewings) of pests on *C. monnieri* by *S. heraclei* might influence their biocontrol ability. The population dynamics and corresponding control strategies for aphid *S. heraclei* on honeysuckle have received increased attention due to their medical value [15,16,17,18]. Currently, studies on the adaptability of aphid *S. heraclei* to functional plant *C. monnieri* are still limited.

The host plant quality, including its defensive compounds and physical properties, significantly influences the performance of herbivorous insects. Long-term interaction between aphid feeding habits and host plants involves a range of induced, reciprocal, and responsive mechanisms. Some species of aphids feed on a variety of host plants, but most species of aphids are specialized or even highly specialized to one specific host plant or different individuals of the same host plant species, exhibiting variable fitness levels depending on the host plant species [19]. Aphid fitness, including the developmental time, fecundity, and average lifespan, have been shown to vary among genetically distinct clonal lines in response to plant stress [20], different host plants [21], and feeding experience [22]. Additionally, leaf surface characteristics, nutrient content, and plant age often influence aphid feeding behavior on host plants [23]. Some *Serratia* genera not only positively influence plant quality but also suppress the plant defense via aphid saliva transmission [24,25]. The interactions of endosymbionts and host plants influence the feeding behavior of aphids [26].

Symbionts have evolved symbiotic relationships with aphids, profoundly influencing their host’s biology, notably impacting their dietary breadth and fitness [27]. Almost all aphids harbor an obligate endosymbiont, *Buchnera aphidicola* Buchnera (Gammaproteobacteria: Enterobacteriaceae), which provides essential amino acids and enhances the resistance to heat stress [28]. Apart from the obligate endosymbiont, some aphids harbor one or more facultative symbionts. These endosymbionts are not essential for host survival and reproduction and can be inherited both vertically and horizontally [29]. Facultative endosymbionts enhance aphid host performance and fitness under external conditions by altering amino acid requirements, shortening development times, and improving fecundity [30,31,32]. Facultative endosymbiont *Serratia symbiotica* Moran (Gammaproteobacteria: Enterobacteriaceae) improves the performance of pea aphid *Acyrthosiphon pisum* Harris (Hemiptera: Aphididae) to withstand unfavorable conditions (e.g., improving heat tolerance, protecting against parasitoids, and mitigating plant defense mechanisms) [33]. However, *S*. *symbiotica* does not affect adult body mass or fecundity but increases nymph weights and temporarily boosts *B. aphidicola* titers; it also inhibits winged-to-wingless morph regression to enhance its spread [34]. When introduced to plants, cultured *S*. *symbiotica* colonizes the tissue, mediates horizontal transfer among aphids, and enhances plant growth, most notably in the root [24]. Currently, little research has been conducted on endosymbionts of the aphid *S. heraclei*.

The aphid *S. heraclei*, primarily feeding on Apiaceae and Caprifoliaceae plants, adapts with significant performance variation across different host plants and various aphid clones. *S*. *symbiotica* also plays an important role in aphid fitness. However, the performance of aphid *S. heraclei* on *C. monnieri* and the role of *S. symbiotica* remain largely unexplored. This study investigated the prevalence of facultative endosymbionts in aphid populations collected from *L*. *japonica, C*. *monnieri,* and *A*. *graveolens* through multiplex diagnostic PCR. Four aphid clones were obtained for fitness experiments on original and novel hosts. This study pursued two primary objectives: (1) to systematically characterize the prevalent patterns of facultative endosymbionts in aphid populations of *S. heraclei*, and (2) to evaluate host plant-dependent fitness trade-offs associated with *S. symbiotica*. Our findings advance the mechanistic understanding of tripartite symbiosis dynamics in herbivore–microbe–plant systems.

## 2. Results

### 2.1. Presence of Facultative Endosymbionts of Semiaphis heraclei

**Figure 1:**
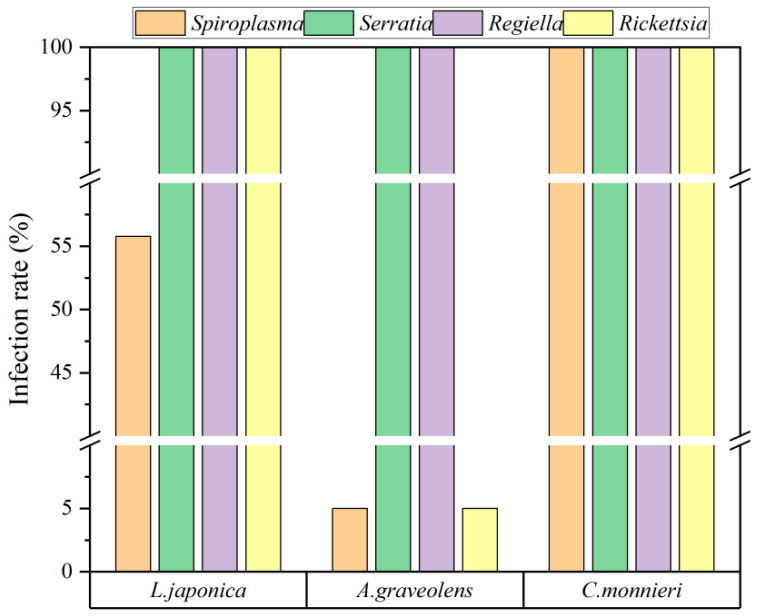
The presence and infection rates of facultative endosymbionts in aphid *S*. *heraclei* on three host plants.

A total of 87 aphid samples were collected from three host plants, namely *L. japonica* (52), *A. graveolens* (20), and *C. monnieri* (15). Four species of facultative endosymbionts, *S. symbiotica*, *Regiella insecticola* Moran (Gammaproteobacteria: Enterobacteriaceae), *Spiroplasma spp.*, and *Rickettsia spp.,* were detected in aphid *S. heraclei* at varying infection rates. Among these, *R. insecticola* and *S. symbiotica* were ubiquitous in all aphid populations, irrespective of the host plants on which they were reared. *Rickettsia* was detected in all aphids, except those on *A. graveolens*, which showed only a 5% infection rate. Among aphids reared on *C. monnieri*, the infection rate of *Spiroplasma* was 100%, whereas those on *L. japonica* and *A. graveolens* had much lower rates of 55.77% and 5%, respectively. A chi-squared test revealed significantly higher infection rates of *Spiroplasma* in aphids feeding on *C. monnieri* compared to those on *L. japonica* (*χ*^2^ = 67.00, *p* < 0.001) or *A. graveolens* (*χ*^2^ = 48.26, *p* < 0.001). Similarly, the infection rate of *Rickettsia* was also higher on *C. monnieri* than on *A. graveolens* (*χ*^2^ = 48.26, *p* < 0.001) (Figure 1).

### 2.2. Developmental Duration of Nymph Stage of S. heraclei on Two Host Plants

We predetermined four aphid clones to confirm the absence or presence of *S. symbiotica* and *R. insecticola* by diagnostic PCR before conducting fitness experiments. As shown in Table 1 and Table 2, aphid clones and host plants significantly affected the nymphal development time and three other development parameters (last molt, last molt to reproduction, and start of reproduction), except for the development time of the second and fourth instars, not being related to the host plant. Their interaction significantly influenced the development time of the first to third instars and the time of last molt and start of reproduction.

**Figure 2:**
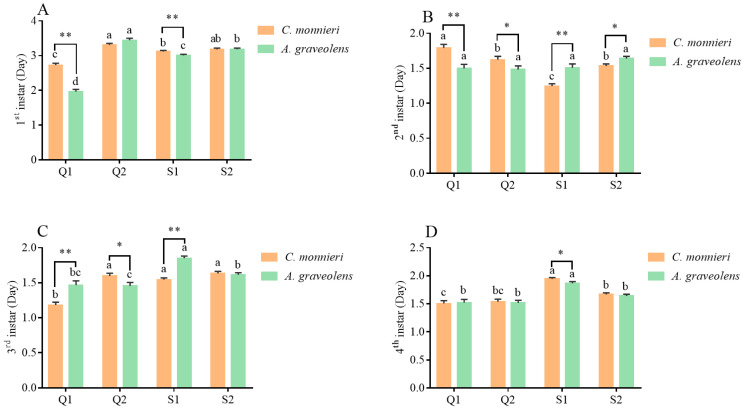
The development durations of aphid *S. heraclei* on two host plants at the stages of the first instar (**A**), second instar (**B**), third instar (**C**), and fourth instar (**D**). Data are represented as the mean ± standard error (SE). Different lowercase letters indicate significant differences in the developmental durations of the four aphid clones on different plants based on Tukey’s HSD test (*p* < 0.05). Statistical significance (*t*-test, *p* < 0.05) between two host plants at the same stage is indicated as follows: * *p* < 0.05, ** *p* < 0.01. No marks indicate no significant difference (*p* > 0.05).

The developmental durations of the nymph stage in the four aphid clones on *C. monnieri* and *A. graveolens* are presented in Figure 2. The nymphal developmental duration varied significantly among aphid clones and nymph instars. Compared to feeding on *A. graveolens*, Q1 clones on *C. monnieri* exhibited longer first-instar (*t* = 8.68, *p* < 0.001) and second-instar durations (*t* = 3.83, *p* < 0.001) but shorter third-instar development (*t* = 3.98, *p* < 0.001), while Q2 clones showed accelerated development for the second (*t* = 2.05, *p* = 0.043) and third instars (*t* = 2.25, *p* = 0.025). Aphid clone S1 developed more slowly on *C. monnieri* in the first instar (*t* = 2.78, *p* = 0.006) and fourth instar (*t* = 2.20, *p* = 0.03), but faster in the second instar (*t* = 4.16, *p* < 0.001) and third instar (*t* = 7.67, *p* < 0.001), compared to that on *A. graveolens*. Aphid clone S2 took less time only in the second instar (*t* = 2.57, *p* = 0.011) on *C. monnieri*.

Regarding first-instar development, Q1 aphid clones exhibited the shortest duration on both host plants (*C. monnieri*: *F*_3, 225_ = 33.79, *p* < 0.001; *A. graveolens*: *F*_3, 209_ = 173.63, *p* < 0.001), followed sequentially by S1, S2, and Q2. At the second instar on *C. monnieri*, Q1 showed the longest development (*F*_3, 225_ = 32.36, *p* < 0.001), while S1 had the shortest. At the third instar on *C. monnieri*, Q1 exhibited the shortest development time among all aphid clones (*F*_3, 225_ = 39.21, *p* < 0.001). On *A. graveolens*, Q2 developed the fastest in comparison to S1 and S2 (*F*_3, 209_ = 19.49, *p* < 0.001). In contrast, S1 took the longest time to reach the fourth instar on both *C. monnieri* (*F*_3, 225_ = 28.01, *p* < 0.001) and *A. graveolens* (*F*_3, 209_ = 16.44, *p* < 0.001).

**Figure 3:**
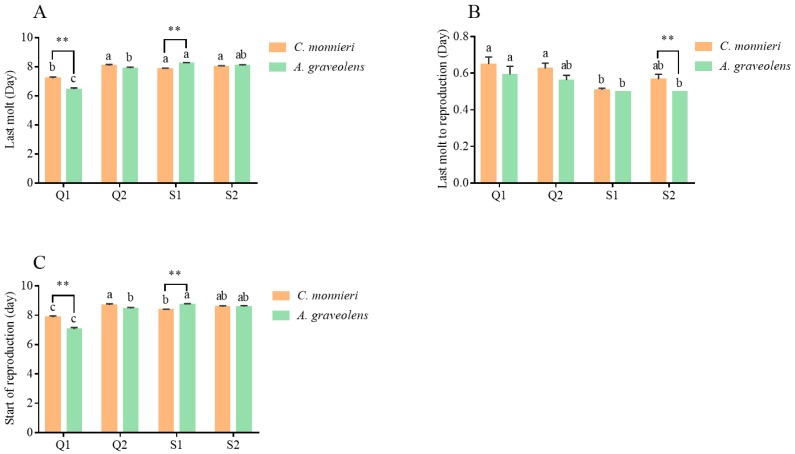
Development parameters, i.e., last molt (**A**), last molt to reproduction (**B**), and start of reproduction (**C**), of aphid *S. heraclei* on two host plants. Data are represented as the mean ± standard error (SE). Different lowercase letters indicate significant differences in the three parameters among the four aphid clones based on Tukey’s HSD test (*p* < 0.05). Statistical significance (*t*-test, *p* < 0.05) between two host plants at the same stage is indicated as follows: ** *p* < 0.01. No marks indicate no significant difference (*p* > 0.05).

As shown in Figure 3, whenever reared on *C. monnieri* and *A. graveolens*, the aphid clone Q1 exhibited the shortest time to last molt (*p* < 0.001) and to have offspring (*p* < 0.01) but the longest time from last molt to reproduction (*p* < 0.001) compared to the other three aphid clones. Furthermore, when reared on *A. graveolens*, Q1 developed faster than on *C. monnieri* (*p* < 0.001), while the opposite trend was observed for aphid clone S1 (*p* < 0.001). Aphid clone S2 reared on *C. monnieri* exhibited a longer time from last molt to reproduction (*p* < 0.05). *S*. *symbiotica* prolonged the development time in aphids collected from *A*. *graveolens*. Additionally, aphids developed faster when feeding on their natal host plants.

### 2.3. Performance of Different Aphid Clones on Different Host Plants

**Figure 4:**
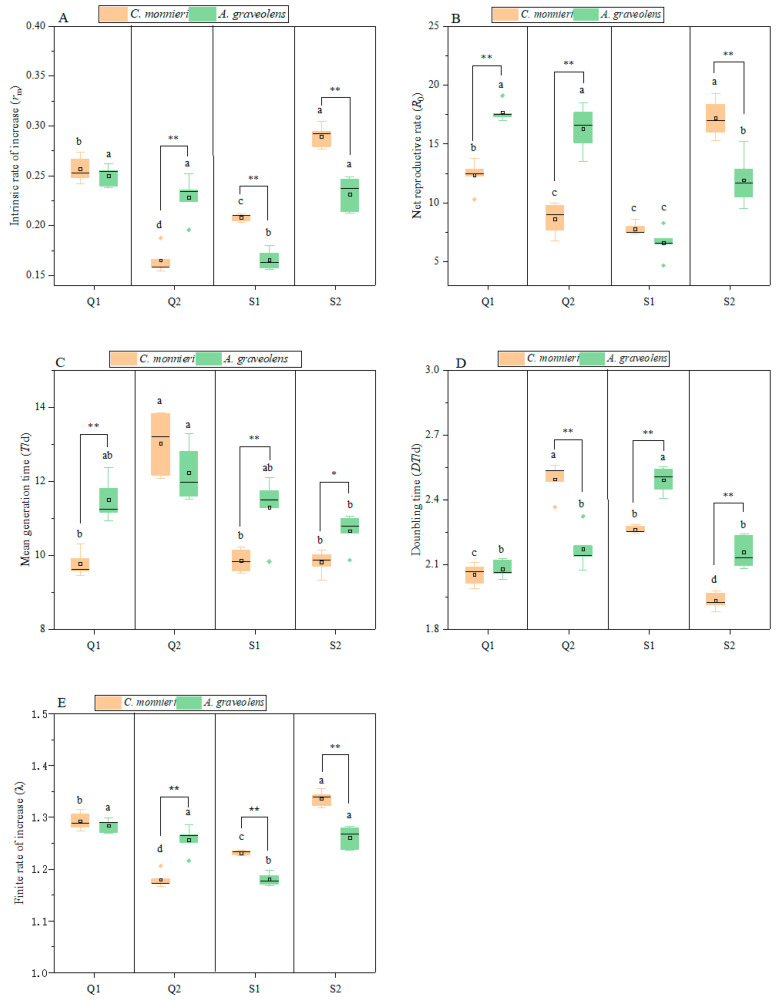
Life table parameters of four aphid clones on two host plants. Intrinsic rate of increase (**A**). Net reproductive rate (**B**). Mean generation time (**C**). Doubling time (**D**). Finite rate of increase (**E**). Data are represented as the mean ± standard error (SE). Different lowercase letters indicate significant differences in the five life table parameters of the four aphid clones on different plants based on Tukey’s HSD test (*p* < 0.05). * *p* < 0.05, ** *p* < 0.01. No marks indicate no significant difference (*p* > 0.05).

The life table parameters, including the intrinsic rate of increase (*r*_m_), net reproductive rate (*R*_0_), mean generation time (*T*), doubling time (*DT*), and finite rate of increase (λ), were significantly affected by the host plant, aphid clone, and their interaction, although the doubling time remained unaffected by the host plant (Table 3). All five parameters varied significantly across aphid clones and host plants (Figure 4). On *C. monnieri*, *r*_m_ varied significantly among clones (*F*_3, 16_ = 119.44, *p* < 0.001), ranking as follows: Q2 (0.17) < S1 (0.21) < Q1 (0.26) < S2 (0.29). On *A. graveolens*, *r*_m_ also differed significantly (*F*_3, 16_ = 28.42, *p* < 0.001), and that of S1 was lower than that of Q2, S2, and Q1. Moreover, *r*_m_ was higher on *C. monnieri* than on *A. graveolens* for clones S1 and S2 (*p* < 0.001) but lower for Q2 (*p* < 0.001). As for the net reproductive rate (*R*_0_), on *C. monnieri*, *R*_0_ differed significantly (*F*_3, 16_ = 55.99, *p* < 0.001), ranking as follows: S1 (7.8) < Q2 (8.66) < Q1 (12.36) < S2 (17.2). On *A. graveolens*, *R*_0_ differed significantly (*F*_3, 16_ = 44.06, *p* < 0.001), with that of S1 (6.61) being lower than that of S2 (11.96), Q2 (16.28), and Q1 (17.7). *R*_0_ was lower on *C. monnieri* than on *A. graveolens* for Q1(*t* = 7.83, *p* < 0.001) and Q2 (*t* = 6.99, *p* < 0.001), but higher for S2 (*t* = 4.24, *p* = 0.003).

As regards the mean generation time (*T*), when aphids were reared on *C. monnieri*, *T* differed significantly among clones (*F*_3, 16_ = 47.96, *p* < 0.001), with that of Q2 being higher than that of S1, S2, and Q1. On *A. graveolens*, *T* differed significantly among clones (*F*_3, 16_ = 4.38, *p* = 0.02), especially between S2 and Q2. *T* was shorter on *C. monnieri* than on *A. graveolens* for S1 (*t* = 3.43, *p* = 0.009), S2 (*t* = 3.31, *p* = 0.011), and Q1 (*t* = 5.74, *p* < 0.001), but not for Q2. On *C. monnieri*, *DT* differed significantly (*F*_3, 16_ = 116.26, *p* < 0.001), ranking as follows: S2 < Q1 < S1 < Q2. However, *λ* showed the opposite (*F*_3, 16_ = 117.2, *p* < 0.001). On *A. graveolens*, *DT* differed significantly (*F*_3, 16_ = 32.93, *p* < 0.001), with S1 > Q1, Q2, and S2. *λ* differed significantly (*F*_3, 16_= 27.47, *p* < 0.001), with S1 < S2, Q1, and Q2. The *DT* of S1 (*t* = 7.96, *p* < 0.001) and S2 (*t* = 5.82, *p* < 0.001) reared on *C. monnieri* was lower than in that reared on *A. graveolens,* but the opposite was observed for the finite rate of increase (λ) (*p* < 0.001). Similarly, the *DT* of Q2 reared on *C. monnieri* was higher than in that on *A. graveolens* (*t* = 5.95, *p* < 0.001), while *λ* showed the opposite trend (*t* = 5.7, *p* = 0.001).

## 3. Discussion

### 3.1. Facultative Endosymbiont Dynamics in Aphid S. heraclei

To our knowledge, this is the first study to investigate the endosymbiont communities and their infection rates in the aphid *S*. *heraclei* across three host plants, *A. graveolens*, *C. monnieri*, and *L. japonica*. Interestingly, our results indicated that there were no significant differences in the community structure of facultative endosymbionts among aphids from different host plants.

Notably, four facultative endosymbiont species, *S. symbiotica*, *Spiroplasma spp*., *Rickettsia spp*., and *R. insecticola*, were detected in aphid *S. heraclei*. Among these, the infection frequencies of *Spiroplasma* and *Rickettsia* varied significantly across host plants, whereas those of *S. symbiotica* and *R. insecticola* were host-independent. This contrasts with broader aphid endosymbiont trends [35], where facultative symbiont communities exhibit considerable variation, with *S. symbiotica* being the most prevalent (47% of 156 aphid species) and *Arsenophonus* Hertig. (Gammaproteobacteria: Enterobacteriaceae) the least (7% of 131 aphid species). In the present study, *S*. *symbiotica* and *R. insecticola* were ubiquitous in aphid *S. heraclei* from three host plants (*L. japonica*, *A. graveolens*, and *C. monnieri*), while other facultative symbionts, including *Spiroplasma* and *Rickettsia*, exhibited significant variation among different aphid populations. Conversely, cotton aphid *A. gossypii* exhibits a high prevalence in facultative endosymbionts across China, with six species identified among 34 species of host plants [36]. In particular, frequencies of up to 98% were detected for *Arsenophonus* in the aphid *A. gossypii* on Caprifoliaceae, Polygonaceae, and Rosaceae plants [36]. As such, the conserved infection pattern observed here suggests that *S. symbiotica* and *R. insecticola* may play particularly stable and essential roles in aphid *S. heraclei*, possibly reflecting co-adaptive evolution or specific ecological constraints.

The diversity and prevalence of facultative endosymbionts in aphids varied with multiple factors, including the host plant species, geographical location, season, and external temperature. The infection rate of *Arsenophonus* was higher in aphid *A. gossypii* feeding on cotton than in aphids on cucumber [31]. Comparative studies revealed a greater diversity of endosymbionts in aphid *A. gossypii* sampled from Xinjiang than in those from Henan; regional variations are also evident in the infection frequencies, as aphids from Hainan showed higher prevalences of *Hamiltonella defensa* Moran (Enterobacteriales: Enterobacteriaceae) and *S. symbiotica*, while those form Xinjiang exhibited a greater incidence of *Arsenophonus* [37]. Additionally, the facultative endosymbionts in cotton aphid *A. gossypii* exhibited obvious seasonal dynamics; for example, the *Arsenophonus* infection rates were lower in summer than in autumn [21]. The infection rates and abundances of endosymbionts in aphids decreased under inappropriate temperatures, with some symbionts being eliminated when exposed to a lower or higher temperature [21,38]. A similar seasonal pattern of *Arsenophonus* infection has been reported in honeybees, with the prevalence gradually increasing from spring to autumn but being lost during overwintering [39]. In the aphid *Adelges tsugae* Annand (Hemiptera: Adelgidae), while the overall prevalence of *S. symbiotica* was higher in Georgia than in New York, no significant differences were observed at finer spatial scales, within individual trees or plots or even across sampling sites within the same state [40]. This suggests that factors other than local selection may shape symbiont distribution. Notably, *S. symbiotica* has transitioned from a facultative to a co-obligate symbiont in certain aphid lineages [41,42], highlighting its evolving ecological role. Moreover, the infection frequency and distribution of facultative endosymbionts can be influenced by non-selective factors such as the transmission efficiency, host migration, and genetic drift [43]. The generalizability of our findings on the *S. heraclei* endosymbiont community, particularly the high prevalence of *S. symbiotica*, is limited by the singular geographical (Jinan) and temporal (April) scope of our sampling. To ascertain whether this pattern is attributable to functional traits, the local climate, or seasonal chance, future work must be expanded to include multi-seasonal and multi-geographic sampling strategies.

### 3.2. Differentiation of Aphid Host Adaptability

Some characteristics of host plants, including nutrients, secondary metabolites, and morphology, could significantly influence the development and reproduction of herbivorous insects. Previous studies on aphid *S. heraclei* (originating from *L. japonica* and *Glehniae radix*) have demonstrated host-dependent variations in fitness parameters. For example, two populations of *S. heraclei* exhibited significant differences in the total developmental duration and fecundity when reared on five Apiaceae plants (*Angelica dahurica* Fisch (Apiaceae), *A. graveolens*, *Bupleurum chinense* Franch. (Apiaceae), *Glehniae radix* (Apiaceae), *L. japonica*) [15]. Comparable differences were observed across other Apiaceae hosts, including *Coriandrum sativum* L. (Apiaceae), *Daucus carota* L. (Apiaceae), and *A. graveolens* [2]. Consistent with these findings, our study revealed host adaptability differentiation among four populations of *S. heraclei* reared on two Apiaceae plants (*C. monnieri*, *A. graveolens*). Aphids performed better on their natal host plants, exhibiting higher fecundity and shortening nymphal development. Additionally, the presence of *S. symbiotica* improved the performance of aphids collected from plant *C. monnieri*. However, it decreased the fecundity of aphids collected from plant *A. graveolens* only on *C. monnieri*.

Adaptive differences are likely to be mediated by various plant nutrients and secondary metabolites [21,25,31,44]. The presence of facultative endosymbionts is closely related to the performance of aphids on host plants. For instance, the presence of *Aresnophonus* improved aphid fitness when feeding on an artificial diet with plant secondary metabolite gossypol, and the population density of *Arsenophonus* increased as the concentration rose [21]. In addition, plant hormones, such as salicylic acid (SA) and indole acetic acid (IAA), influenced the fitness parameters of aphid *S. heraclei* when sprayed on host plant *C. monnieri* [44]. Such aphid–plant interactions might lead to host adaptability differentiation and even specialization for one or more host plant species. This phenomenon has occurred in many species of aphids, including pea aphid *A. pisum*, *A. gossypii*, *Schizaphis graminum* Rondani (Hemiptera: Aphididae), *Sitobion avenae* Fabricius (Hemiptera: Aphididae), and so on [25,45,46,47]. Commonly, specialized aphid clones exhibit optimal performance on one host plant but perform poorly on alternative hosts. In the present study, the aphids collected from plant *C. monnieri* exhibited better performance on their natal host *C. monnieri* than on alternate host *A. graveolens* and vice versa. Some facultative endosymbionts are related to the host range of aphids, affecting the host’s specialization. Our findings suggest that the role of *S. symbiotica* in aphid fitness is related to the aphid origin and host plant. Nevertheless, the mechanisms underlying this adaptability differentiation in *S. heraclei*, and whether host specialization has evolved, require further investigation.

### 3.3. Multiple Effects of Serratia symbiotica on Aphid Fitness

Infection with facultative endosymbionts confers context-dependent fitness benefits to aphids, varying with the host plant, aphid clone, and symbiont strain. While the predominant literature emphasizes the nutritional and protective roles of *S. symbiotica*, a notable body of research presents contrasting findings. For instance, studies utilizing cultured *S. symbiotica* strains have demonstrated that infection reduced development, reproduction, and body weight in the pea aphid *A. pisum* but increased the susceptibility to certain insecticides [48]. In the case of aphid *S. heraclei*, our study provides the first investigation of host plant-dependent variations in facultative endosymbiont communities and infection dynamics. Symbionts conferring fitness benefits to their hosts tend to establish persistently high infection rates within host populations, often leading to prevalence [22]. Fitness enhancements, including accelerated development and increased reproductive output, facilitate population expansion. In the present study, an exceptionally high prevalence of *S. symbiotica* was found in the aphid *S. heraclei* across aphid populations on diverse host plants. Critically, an increase in the fecundity of the aphid harboring *S. symbiotica*, together with the acceleration of nymphs, facilitated the population’s expansion, aligning with findings in other aphids [49,50,51] and demonstrating its significant role in plant–insect interactions. Meanwhile, some studies report negative fitness effects for facultative endosymbionts (e.g., *H. defensa* and *R. insecticola*) depending on the host/symbiont strains, host plants, and environment [52], or neutral effects of *S. symbiotica* in the pea aphid *A. pisum* [34,52]. Our findings suggest that *S. symbiotica* has broad potential to provide fitness benefits to a wide range of aphid populations due to the widespread context-dependent advantages. *S. symbiotica* enhances aphid growth by increasing fatty acid synthesis and triacylglycerol storage [50] or by providing essential amino acids [48], as demonstrated in *Cinara cedri* Mimeur (Hemiptera: Aphididae). Additionally, it can metabolize diverse carbon sources from its host [48]. *S. symbiotica* enhances host plant feeding by manipulating salivary gland gene expression to inhibit plant defenses [53]. Further investigation is required to elucidate the precise roles of *S. symbiotica* in aphids and its underlying mechanisms. In particular, the mechanisms through which *S. symbiotica* modulates the fitness of *S. heraclei* warrant further investigation.

### 3.4. Co-Adaptation in Aphid–Plant Associations Mediated by Serratia symbiotica

Strong correlative associations exist between specific facultative endosymbionts and aphid populations adapted to host plants [54]. Our study provides further evidence for this view, demonstrating that the association with *S. symbiotica* in *S. heraclei* is not fixed but is a context-dependent relationship influenced by the aphid origin and host plant. This suggests that the functional significance of *S. symbiotica* is shaped by a complex interplay among the host genotype and ecological factors.

The context-dependent effect appears to be general for facultative endosymbionts. The presence of *Arsenophonus* alters the aphid’s requirement for amino acids, leading to lower infection rates in aphids feeding on cucumber, with high levels of amino acids [31]. Moreover, *Arsenophonus* enhances aphid fitness on diets containing the plant secondary metabolite gossypol, with its population density increasing in line with the gossypol concentration [21]. Furthermore, the ability of *Arsenophonus* to modulate the dietary breadth of *A. gossypii* by enhancing nutrient synthesis in the obligate symbiont *B. aphidicola* improved host specialization on cotton [55].

Fitness assessment revealed that *S. symbiotica* infection did not contribute to the performance of aphids collected from plant *A. graveolens* on *C. monnieri*. Conversely, infection with *S. symbiotica* enhanced the performance of aphids collected from plant *C. monnieri* on both host plants. These host-specific effects align with previous findings [56]. We speculate that the role of *S. symbiotica* is not universal but is related to the aphid origin and the quality of host plants. Therefore, the potential influences of aphids’ genetic background and host plant identity on facultative endosymbiont-mediated fitness outcomes cannot be overlooked. This view is supported by early studies employing limited strain diversity, which revealed that *S. symbiotica* reduced fecundity in only one of three pea aphid clones tested on *Lathyrus odoratus* L. (Fabales: Fabaceae) and *Medicago polymorpha* L. (Fabales: Fabaceae) [57], highlighting context-dependent outcomes.

Our findings, which demonstrate a clear contrast in the roles of *S. symbiotica* between aphids of different origins, reinforce a co-evolutionary arms race. The superior performance of aphids collected from plant *C. monnieri* when infected with *S. symbiotica* suggests a co-adapted relationship in their native ecological context. Future work is needed to dissect the molecular and physiological mechanisms underpinning this tripartite interaction.

## 4. Materials and Methods

### 4.1. Aphid Samples for Identification of Facultative Symbionts

Aphids were sampled at two fields in Shandong, China: one was at the *C. monnieri* field at the Experimental and Demonstration Base of Shandong Academy of Agricultural Sciences in Jiyang (117°1′27″ E, 36°56′40″ N); the other was at the experimental base of *L. japonica* and *A. graveolens* in Jinan (117°5′12.350″ E, 36°45′22.172″ N). One aphid was sampled every 5 m in one transect, with 10 m between transects. Sampled aphids, whose information is listed in Table 4, were placed in 1.5 mL centrifuge tubes containing 1.0 mL absolute alcohol and then stored at −20 ℃ until DNA extraction. As species-specific primers were shown to be very reliable in detecting the symbiont species in an aphid [58], the symbiont pattern statuses of all aphid strains were periodically characterized by using a multiplex diagnostic PCR method.

### 4.2. Aphid DNA Extraction and Facultative Endosymbiont Detection

A total of 87 aphid samples from *C. monnieri*, *L. japonica*, and *A. graveolens* were used for the identification of facultative endosymbionts. Total aphid genomic DNA was extracted following the methods found in Chang et al. (2022) [21], with minor modification. One aphid was washed with aseptic double-distilled water and ground at 30 Hz for 2 min with two 2 mm zirconia beads using a tissue lyser (TissueLyser II, Retsch GmbH, Hann, Germany) with 50 μL TE buffer (pH = 8.0, Sangon Biotech, Shanghai, China) in an aseptic centrifuge tube. We added 2 μL 20 mg/mL proteinase K (Vazyme, Nanjing, China) and centrifuged the samples at 6000 rpm for 1 min at room temperature using a centrifuge (Eppendorf 5425R, Hamburg, Germany). The homogenate was subsequently incubated at 37 °C for 30 min using an electro-thermostatic water bath (DK-8D-3J, Guangdong, China), followed by 95 °C for 5 min.

All DNA samples were screened for the nine known secondary symbionts using the specific PCR primers listed in Table 5. The 15 μL PCR reaction system contained 7.5 μL 2 × Rapid Taq Master Mix (Vazyme, Nanjing, China), 4 μL ddH_2_O, 1 μL forward/reverse primer (Sangon Biotech, Shanghai, China), and 1.5 μL sample DNA. The PCR reaction procedures were performed at 94 ℃ for 5 min followed by 35 cycles of 94 ℃ for 15 s, 72 ℃ for 30 s, and 72 ℃ for 10 min for the final extension, being finally held at 4 ℃. Finally, the amplification products were detected by 2% agarose gel electrophoresis and stained with GeneRed nucleic acid dye (RT211, Tiangen, Beijing, China) using the DL 2000 DNA marker (3427A, Takara, Dalian, China). The detection of target amplification products indicated aphid infection with the target facultative symbiont. Conversely, the absence of these specific products confirmed that the aphids were uninfected.

**Table 5:** Sequences of specific primers for nine facultative endosymbionts.

Facultative Symbiont	Primer	Sequence 5′-3′	Tm/°C	Product Size/bp	Reference
*Arsenophonus*	fbaAF	GCYGCYAAAGTTCRTTCTCC	52	617	[59]
	fbaAR	GGCAAATTAAATTTCTGCGCAACG			[60]
*Wolbachia*	wspF	GGGTCCAATAAGTGATGAAGAAAC	56	570	[61]
	wspR	TTAAAACGCTACTCCAGCTTCTGC			
*Spiroplasma spp.*	16SA1	AGAGTTTGATCMTGGCTCAG	55	510	[62]
	TKSSsp	TAGCCGTGGCTTTCTGGTAA			[63]
*S.* *symbiotica*	10F	AGTTTGATCATGGCTCAGATTG	55	480	[64]
	R443R	CTTCTGCGAGTAACGTCAATG			[65]
*Rickettsia spp.*	16SA1	AGAGTTTGATCMTGGCTCAG	55	200	[62]
	16SR	CATCCATCAGCGATAAATCTTTC			[66]
*H.* *defensa*	10F	AGTTTGATCATGGCTCAGATTG	50	471	[64]
	T419R	AAATGGTATTSGCATTTATCG			[65]
*Rickettsiella spp.*	P136F	GGGCCTTGCGCTCTAGGT	55	300	[67]
	P136R	TGGGTACCGTCACAGTAATCGA			
*R.* *insecticola*	10F	AGTTTGATCATGGCTCAGATTG	55	200	[64]
	U433R	GGTAACGTCAATCGATAAGCA			[65]
PAXS	PAXSF PAXSR	AGTTTGATCATGGCTCAGATTG	55	500	[68]
		GCAACACTCTTTGCATTGCT			

### 4.3. Aphid Clones and Rearing

The aphid *S. heraclei* colony was obtained from a laboratory culture reared from the progeny of a single apterous aphid. It was collected from *C. monnieri* and *A. graveolens* in the field through the identification of symbionts by PCR; the sampling locations were as described in Section 4.1. Two aphid clones (S1, S2) collected from plant *C. monnieri* and two clones (Q1, Q2) collected from plant *A. graveolens* were continuously maintained for more than 20 generations on Petri dishes containing their respective original host plant leaves on 1% agar (Table 6). Aphids were maintained under standardized conditions (25 ± 1 °C, 75% ± 5% RH, 14L:10D photoperiod) [2,69], with fresh leaves replaced triweekly to ensure optimal conditions.

### 4.4. Aphid Performance Measurement

**Figure 5:**
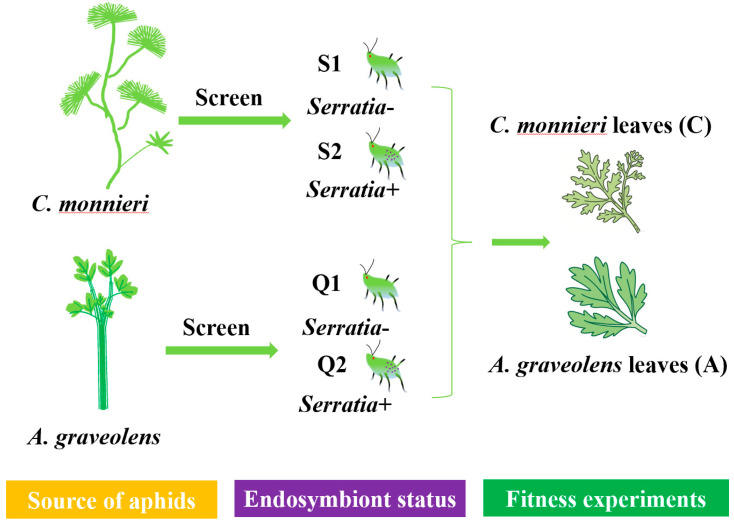
Experimental design for the performance of *S. heraclei* on two Apiaceae plants. Note: Two aphid clones (S1—*Serratia*-, S2—*Serratia*+) and two aphid clones (Q1—*Serratia*-, Q2—*Serratia*+) were, respectively, screened from *C. monnieri* and *A. graveolens* through multiplex diagnostic PCR. Then, these four aphid clones were transferred onto the leaves of *C. monnieri* and *A. graveolens* for fitness assessment, as described in the main text.

The experimental design for fitness assays is summarized in Figure 5. To determine the effects of facultative endosymbiont *S. symbiotica* on individual performance, a uniform-sized 7-day-old wingless aphid was transferred onto a detached leaf of *A. graveolens* or *C. monnieri* on 1% agar in a 30 mm Petri dish. After two hours (post-reproduction onset), all but one newborn nymph were moved to standardize the starting point. Molting processes were recorded at 12 h intervals. Nymphs were tracked using exuviae removed from each Petri dish. For each aphid clone, a minimum of 50 nymphs were tracked to measure their development times and developmental parameters from birth to reproduction. To maintain optimal conditions, the fresh leaves were also replaced every three days.

Aphid fitness indices, including the net reproductive rate (*R*_0_), mean generation time (*T*), intrinsic rate of increase (*r*_m_), doubling time (*DT*), and finite rate of increase (*λ*), were measured by establishing a time-specific life table. Ten 8-day-old adult aphids with a uniform size were transferred onto the leaves of *A. graveolens* and *C. monnieri* in 60 mm Petri dishes containing 1% agar. After 6 h, ten newly born aphids were left in the Petri dishes and served as the initial aphid population. Daily monitoring tracked aphid survival and reproduction until all 10 initial aphids died, with newborns counted and removed upon emergence. Five replicates per aphid clone were conducted, with Petri dishes and excised leaves replaced every three days. Aphid fitness indices were calculated following Liu (2016) [70]. The formulae are as follows, where *x* represents the time; $l_{x}$ represents the survival rate on a particular day *x*; and $m_{x}\;$ represents the number of offspring produced by each aphid:

$$R_{0}=\sum l_{x}m_{x}$$

$$T=\frac{\sum \left(xl_{x}m_{x}\right)}{\sum (l_{x}m_{x})}$$

$$r_{m=}\frac{lnR_{0}}{T}$$

$$DT=ln2/r_{m}$$

$$\lambda =e^{r_{m}}$$

### 4.5. Statistical Analysis

Chi-squared (*χ*^2^) tests were used to assess the differences in facultative endosymbiont infection frequencies across different hosts. Two-way ANOVAs were also used to analyze the effects of aphid clones, host plants, and their interactions on the developmental parameters and life table parameters of aphid *S. heraclei* following host transfer. In addition, the differences in the developmental times of each nymph instar, and the developmental parameters and life table parameters of the aphids among the four aphid clones, were analyzed using one-way analysis of variance (ANOVA) (by using the HSD test at α = 0.05) and an independent *T* test at α = 0.05. All data were processed in SPSS v.26.0.0 (IBM, New York, NY, USA) and all figures were plotted with Origin 2024 (OriginLab Corporation, Northampton, MA, USA) or Graphpad Prism 9.5.1 (GraphPad Software, San Diego, CA, USA).

## 5. Conclusions

Our study provides the first investigation of facultative endosymbiont dynamics in aphid *S. heraclei* across different host plants, with a specific focus on the role of *S. symbiotica*. *Serratia symbiotica* was highly prevalent in aphid *S. heraclei*, suggesting the strong host plant-mediated regulation of this symbiosis. Infection with *S. symbiotica* was associated with significant fitness advantages for aphids collected from *C. monnieri* on their native hosts, but with reduced performance in aphids collected from *A. graveolens* feeding on *C. monnieri*. The consistent outperformance of aphids on their natal host plants, together with the mediating role of *S. symbiotica*, suggests that host plant specialization is mediated by co-adaptation between *S. symbiotica* and the host plant. Furthermore, the superior performance of aphids on their respective natal hosts across all groups points to the evolutionary significance of host–endosymbiont co-adaptation.

## Figures and Tables

**Table 1:** Effects of aphid clones, host plants, and their interactions on developmental time of *S. heraclei*.

Factor	*df*	1st Instar		2nd Instar		3rd Instar		4th Instar	
		*F*	*p*	*F*	*p*	*F*	*p*	*F*	*p*
Aphid clones	3	146.07	<0.001	12.12	<0.001	34.33	<0.001	39.01	<0.001
Host plants	1	32.63	<0.001	1.60	0.203	8.58	<0.001	1.27	0.28
Aphid clones × host plants	3	28.48	<0.001	13.68	<0.001	16.69	<0.001	0.43	0.73

Note: “Aphid clones × host plants” in the fourth line indicates an effect of the interaction between the two factors, aphid clones and host plants, on the developmental time; *F* indicates a test statistic that measures the ratio of "systematic variation" to "random error variation"; *p* indicates a probability value that assesses the statistical significance of the *F*-value.

**Table 2:** Effects of aphid clones, host plants, and their interactions on the developmental parameters of *S. heraclei*.

Factor	*df*	Last Molt		Last Molt to Reproduction		Start of Reproduction	
		*F*	*p*	*F*	*p*	*F*	*p*
Aphid clones	3	139.620	<0.001	8.358	<0.001	109.935	<0.001
Host plants	1	5.961	0.015	6.946	0.009	9.990	0.002
Aphid clones × host plants	3	22.773	<0.001	0.550	0.648	21.495	<0.001

Note: “Aphid clones × host plants” in the fourth line indicates an effect of the interaction between factors on developmental parameters; *F* indicates a test statistic that measures the ratio of "systematic variation" to "random error variation"; *p* indicates a probability value that assesses the statistical significance of the *F*-value.

**Table 3:** The effects of host plants, aphid clones, and their interactions on the performance of aphid *S. heraclei* following host transfer.

Factor	*df*	Intrinsic Rate of Increase (*r*_m_)		Net Reproductive Rate (*R*_0_)		Mean Generation Time (*T*/d)		Doubling Time (*DT*)		Finite Rate of Increase (λ)	
		*F*	*p*	*F*	*p*	*F*	*p*	*F*	*p*	*F*	*p*
Host plants	1	6.65	0.015	11.99	0.002	17.26	<0.001	4.05	0.053	7.33	0.011
Aphid clones	3	79.51	<0.001	57.72	<0.001	31.42	<0.001	79.20	<0.001	78.85	<0.001
Host plants × aphid clones	3	43.04	<0.001	39.17	<0.001	8.37	<0.001	43.84	<0.001	39.17	<0.001

Note: “Aphid clones × host plants” in the fourth line indicates an effect of the interaction between factors on life table parameters; *F* indicates a test statistic that measures the ratio of "systematic variation" to "random error variation"; *p* indicates a probability value that assesses the statistical significance of the *F*-value.

**Table 4:** Sampling information of aphid *S. heraclei*.

Date	Location	Host Plant	Sample No.
20 April 2023	Jinan	*L. japonica*	52
20 April 2023	Jinan	*A. graveolens*	20
26 April 2023	Jiyang	*C. monnieri*	15

**Table 6:** Sampling information and infected facultative endosymbionts of aphid clones.

Aphid Clone	Host Plant	Date	Location	*S. symbiotica*	*R. insecticola*
S1	*C. monnieri*	26 April 2023	Jiyang	-	+
S2	*C. monnieri*	26 April 2023	Jiyang	+	+
Q1	*A. graveolens*	20 April 2023	Jinan	-	+
Q2	*A. graveolens*	20 April 2023	Jinan	+	+

Note: “+” indicates aphid harboring the endosymbiont; “-” indicates no detectable infection; facultative endosymbionts not listed in the table were not detected in aphids.

## Data Availability

All data are contained within the article. The data used to support the findings of this study are available from the corresponding author upon request.
